# Analysis of the zoonotic tick-borne encephalitis virus (TBEV) in raw milk and dairy products in mountain pastures of the Lombardy region, Italy

**DOI:** 10.3389/fmicb.2024.1462645

**Published:** 2024-10-09

**Authors:** Annalisa Scarazzato, Francesco Righi, Marco Pietro Sommariva, Irene Bertoletti, Giovanni Sala, Franco Paterlini, Paolo Daminelli, Guido Finazzi, Marina-Nadia Losio, Enrico Pavoni

**Affiliations:** ^1^Department of Food Safety, Laboratory of Food Control, Istituto Zooprofilattico Sperimentale della Lombardia e dell’Emilia Romagna, Brescia, Italy; ^2^Istituto Zooprofilattico sperimentale della Lombardia e dell’Emilia Romagna, Bergamo, Italy; ^3^Istituto Zooprofilattico sperimentale della Lombardia e dell’Emilia Romagna, Sondrio, Italy; ^4^Istituto Zooprofilattico sperimentale della Lombardia e dell’Emilia Romagna, Binago, Italy; ^5^National Reference Centre Bovine Milk Quality, Istituto Zooprofilattico Sperimentale della Lombardia e dell’Emilia Romagna, Brescia, Italy; ^6^National Reference Centre for Emerging Risks in Food Safety, Istituto Zooprofilattico Sperimentale della Lombardia e dell’Emilia Romagna, Brescia, Italy

**Keywords:** tick-borne encephalitis, TBEV, raw milk, dairy, goat, cattle, foodborne, real-time RT-PCR

## Abstract

Over the last few decades, tick-borne encephalitis (TBE) has become a growing public health problem in Europe. The tick-borne encephalitis virus (TBEV) is a zoonotic virus that affects the central nervous system (CNS). TBEV has been detected in 27 European countries, and the rise in TBE cases is mainly due to environmental and ecological factors, and factors that increase the risk of human exposure to infected ticks. The infection via the alimentary route is the second most common means of TBEV transmission to humans. Raw milk from infected goats, sheep, or cows has been identified as a source of human food-borne infections. This study aims to gather new information on the prevalence of tick-borne encephalitis virus (TBEV) in raw goat’s and cow’s milk and related raw products in the Lombard Alps (Italy). This is important due to the close proximity of Lombardy to the Triveneto region, where TBE is endemic, and southern Switzerland, where numerous TBEV-positive mammals have been found. Throughout 2023, a passive monitoring plan was implemented on samples delivered for TBEV analyses from the Alpine pastures. In total, 248 specimens including raw milk, raw milk cheese, and butter were tested. This is the first monitoring of food at risk of TBEV transmission in a non-endemic region with evidence of TBEV circulation. Despite testing a wide range of dairy products, no sample tested positive for RNA-TBEV by real-time RT-PCR. Preliminary results suggest that raw milk and raw dairy products do not pose a significant risk of TBEV transmission to humans in the territory of Lombardy.

## 1 Introduction

The tick-borne encephalitis virus (TBEV) is a zoonotic virus belonging to the *Flavivirus* genus within the *Flaviviridae* family. It is lipid-enveloped, has a smooth spherical structure with a diameter of 50 nm, and contains a ssRNA (+) genome of approximately 11 kb (Ličková et al., 2021). TBEV causes tick-borne viral encephalitis (TBE), a vaccine-preventable infectious disease affecting the central nervous system (CNS) with neurological symptoms such as meningitis and encephalitis (Van Heuverswyn et al., 2023).

Phylogenetic analysis has identified three main subtypes of the TBEV: European (TBEV-Eu), Far Eastern (TBEV-FE), and Siberian (TBEV-Sib) (Ličková et al., 2021). These subtypes are primarily differentiated according to antigenic differences, vector competence, geographic distribution, and human pathogenicity (Carpi et al., 2009).

Recently, two additional subtypes, Baikalian (TBEV-Bkl) and Himalayan (TBEV-Him), have been identified (Dai et al., 2018). The TBEV-Eu subtype is transmitted by the TBEV-infected tick *Ixodes ricinus* and is endemic in rural areas and forests of central, eastern, and northern Europe. According to the most recent VectorNet report, *Ixodes ricinus* is distributed across a wide geographical area, spanning from Russia to Scandinavia, North Africa, England, central Europe, Italy, and Spain (Tick Maps, 2023; Wint et al., 2023).

TBEV-FE and TBEV-Sib subtypes are mainly transmitted by the tick *Ixodes persulcatus*; the former is endemic in the Far East of Russia, China, and Japan, while the latter is spread in Siberia and some areas of northeastern Europe (Elbaz et al., 2022).

Global climate changes are contributing to the introduction and spread of tick populations and TBEV to new areas and higher altitudes (Knap and Avšič-Županc, 2015; Garcia-Vozmediano et al., 2020; Salat and Ruzek, 2020). The epidemiological observations suggest that TBEV may be considered an emerging disease, given the evidence of a recent increase in TBEV diversity in Europe (Gonzalez et al., 2022).

Currently, there is no data available on the pathogenesis of TBE at the gastrointestinal level in humans, and it is unclear which part of the gastrointestinal tract is affected after oral TBEV infection (Brockmann et al., 2018).

TBE is a growing public health concern in Europe including Italy. The highest number of European reported cases are in central/northern Europe (Brockmann et al., 2018; Paulsen et al., 2019).

In Italy, TBE is endemic in the Triveneto region (north/east) (Garcia-Vozmediano et al., 2020); however, sporadic cases have recently been reported in Lombardy, Emilia Romagna, and Sicily, indicating the virus is expanding into new regions (Ventura Spagnolo et al., 2018; Alfano et al., 2020).

In November 2022, a male hunter aged 49 years displayed clinical symptoms compatible with TBE, reporting a tick bite in the previous month. A serologic exam for TBEV resulted in positive readings for both IgG and IgM; the patient’s clinical picture improved spontaneously, and he was discharged in December 2022. In May 2023, a chamois (*Rupicapra rupicapra*) with ataxia, muscle tremors, incoordination, and frequent swallowing was subjected to autopsy after death. Molecular investigation of the animal’s brain and a pool of its organs confirmed the presence of TBEV, while serological tests also indicated positivity for the tick-borne encephalitis virus. All the ticks retrieved from the body of the chamois were identified morphologically as adult *I. ricinus* and subjected to TBEV PCR, showing clear positivity (Gaffuri et al., 2024). Both cases were recorded in the province of Bergamo, Lombardy.

Similarly, in January 2024, the carcasses of an ibex *(Capra ibex)* and a deer *(Cervus elaphus)* were found in the woods of the Lecco province, Lombardy, to be TBEV-positive with histological alterations of the CNS. Serological investigations conducted the previous summer on livestock at farms showed TBEV-positive goats that were grazing in the same area where dead wild animals were found (Il Giorno, 2024).

A recent molecular screening of ticks collected in Lombardy revealed the absence of TBEV, although serological analysis conducted between 2021 and 2023 demonstrated the presence of TBE antibodies in wild ungulates from the Lombardy region (Gaffuri et al., 2024).

Illness resulting from infection with European subtype viruses may manifest in two phases. The initial phase, which is characterised by fever, and the subsequent phase, during which neurological disorders of varying severity emerge. The biphasic form is dominant for alimentary TBE and represents approximately 20–30% of all TBEV infections after tick bite; however, the mortality rate is relatively low at 2% (Gritsun et al., 2003; Alfano et al., 2020). The TBEV-FE and TBEV-Sib subtypes, on the other hand, cause more severe symptoms and are associated with a mortality rate of 6 and 40%, respectively (Carpi et al., 2009).

The TBE virus is not transmitted directly from person to person, and vertical transmission (mother to foetus) is not entirely clear (Divé et al., 2020); although the principal route of transmission to humans is via the bite of TBEV-infected ticks, recent studies have indicated that the alimentary route may also be a significant factor in the transmission of the virus.

TBEV transmitted by ticks infects different animals, wild and domestic, including voles (the only proven reservoir), goats, sheep, and cattle (endless hosts), which contribute to maintaining the transmission cycle of the infection (Imhoff et al., 2015).

Goats, sheep, or cattle can excrete the virus through milk causing alimentary TBEV infections in humans due to the consumption of unpasteurised milk or dairy products (Süss, 2011), an increasing trend due to the purported health benefits and better taste of natural products (Balogh et al., 2012).

Consequentially, in endemic areas, outdoor activities in the mountains and wooded areas and consumption of raw milk and unpasteurised dairy products are associated with greater risks of infection (Mansfield et al., 2009).

A majority of cases occur between April and August, which correlates with the highest tick questing and feeding activity (Elbaz et al., 2022); nevertheless, sporadic cases were reported also in December and January even when the lifecycle of ticks is in quiescence (Tagliapietra et al., 2011; Van Heuverswyn et al., 2023). This suggests that foodborne transmission may play a significant role in the transmission of TBEV all year long, both by means of fresh products or seasoned cheese.

In most cases of foodborne TBE, the virus is transmitted through goat’s milk and cheese, but TBEV infection through consumption of sheep’s and cow’s milk has also been reported. Infected domestic ruminants do not show clinical symptoms, but can develop a *viraemia* lasting about a week, which is not sufficient to sustain direct viral transmission to other ticks, but is adequate for the transmission of the virus through milk (Alfano et al., 2020).

Even if data on the incidence of foodborne cases are almost lacking, approximately 1% of all TBE cases are thought to be caused by foodborne TBEV (Ruzek et al., 2019; Alfano et al., 2020).

It should be noted that infections by this route may be more common, as some foodborne TBEV infections are asymptomatic or mildly symptomatic, and may therefore go undiagnosed and unrecorded (Buczek et al., 2022).

Thus far, there has been no evidence of foodborne infection with TBEV in Lombardy, where surveillance is active only in the context of the transmission of the virus from ticks to humans, and data are missing for foods at risk of transmission.

This study aimed to the collection of data to define the prevalence of TBEV in the different sampled matrices at risk of contamination and to indirectly evaluate the prevalence of TBEV across the Lombard Alps in the pasture dairy farms.

The study implemented a 12-month monitoring plan mainly focussed on the May–September period, which is the peak season for mountain pasture activities.

## 2 Materials and methods

### 2.1 Sampling

**Figure 1:**
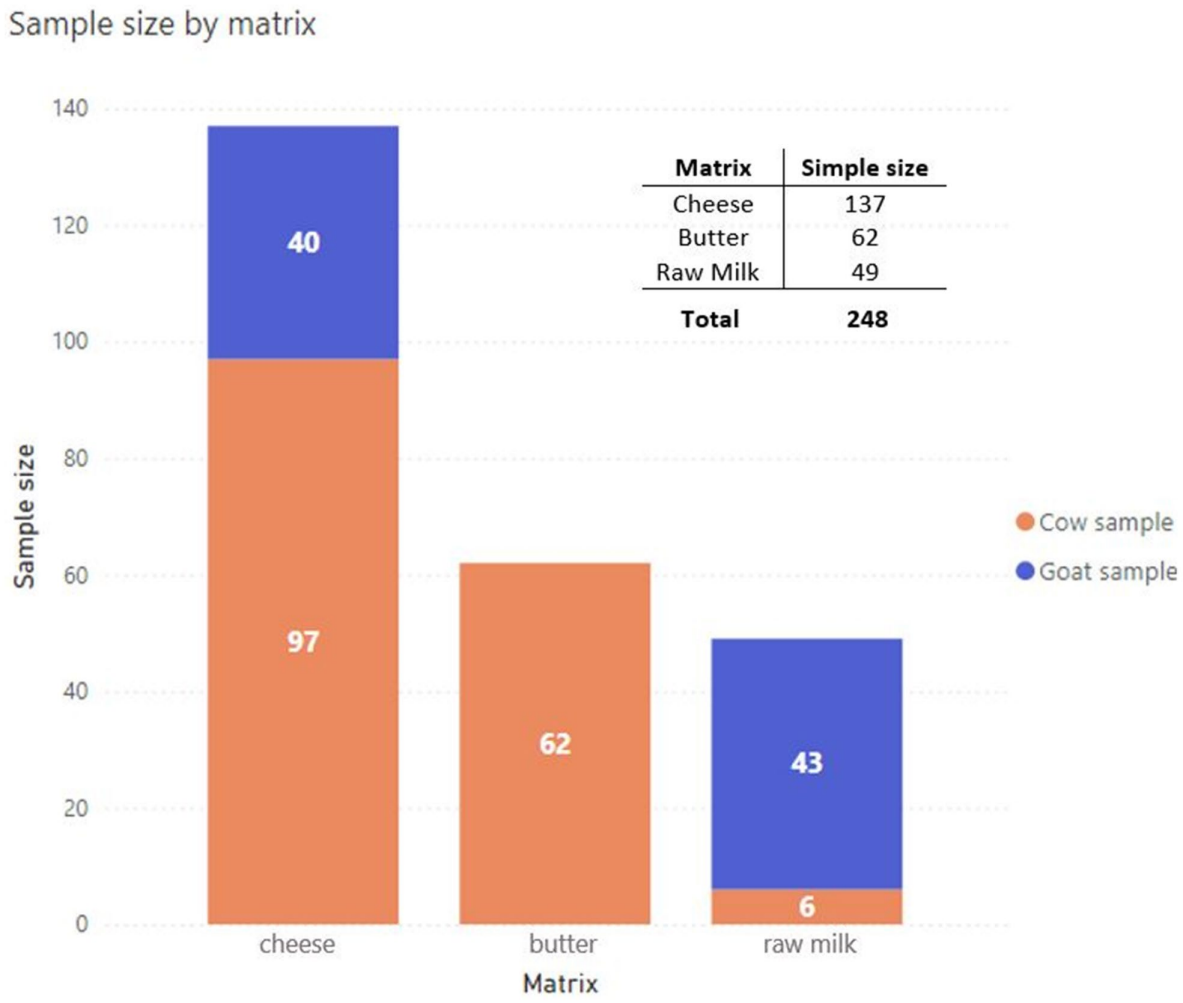
Summary of raw milk and raw milk products collected during the study from January to December 2023 (Power BI, n.d.).

**Figure 2:**
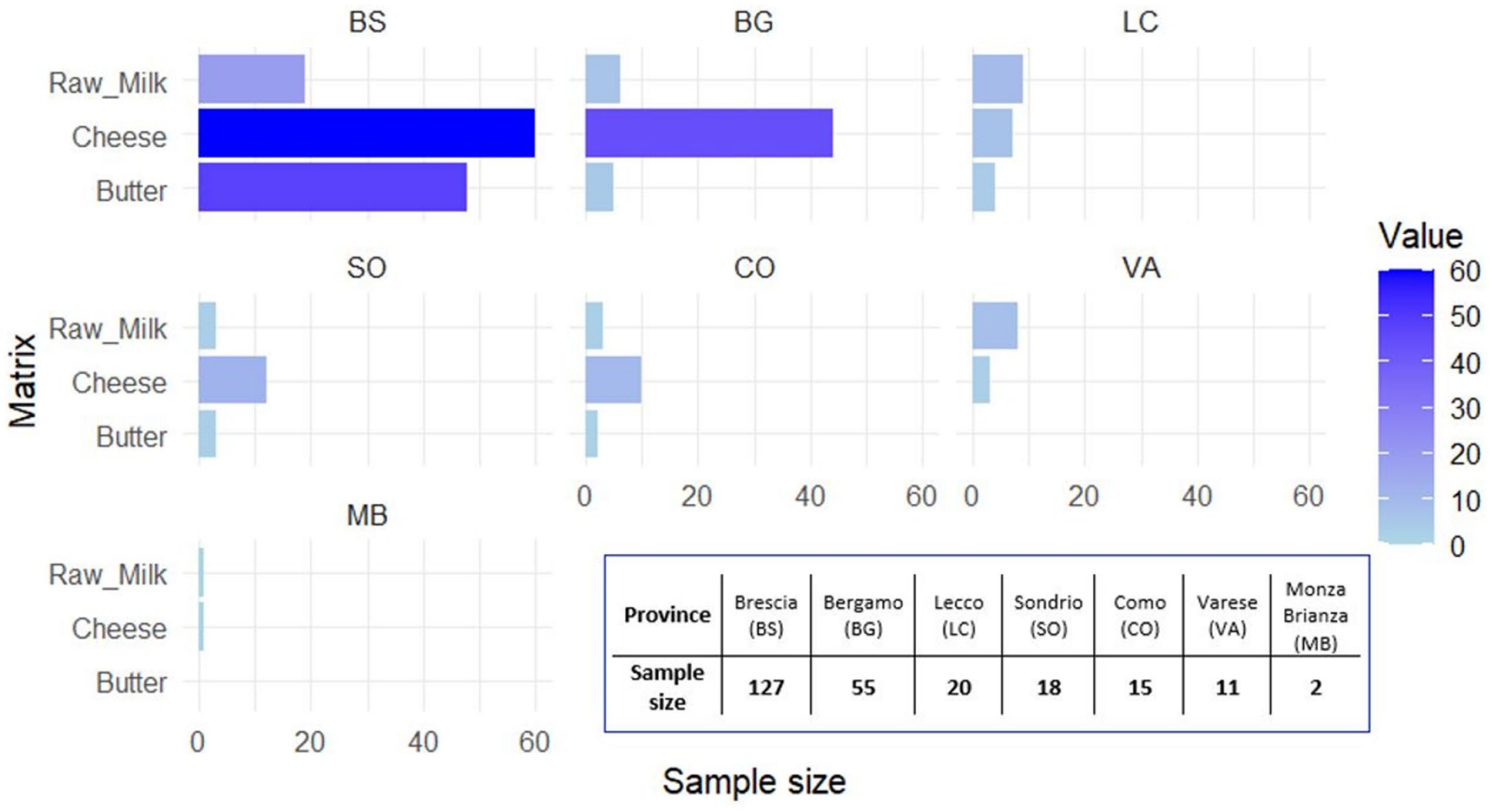
Number of samples collected per province on the Lombardy Alps (R: The R Project for Statistical Computing, n.d.).

**Figure 3:**
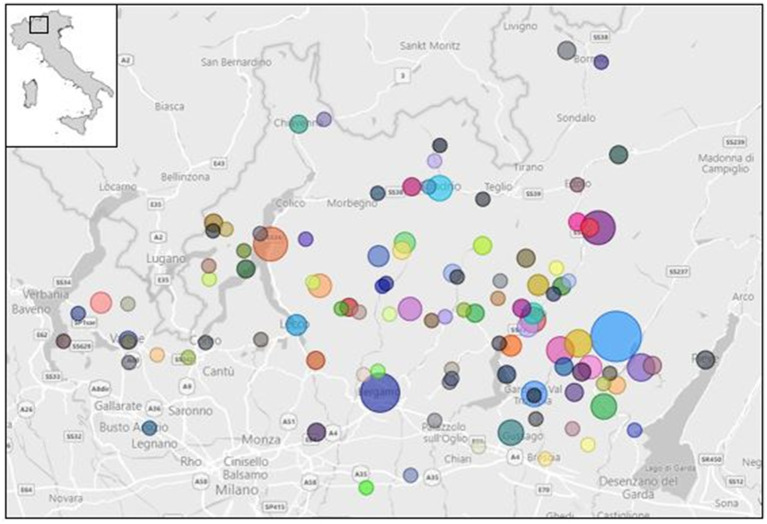
Sampling size and collection spots in Lombardy region (Power BI, n.d.).

From January to December 2023, a total of 248 food samples were collected and analysed, which included raw/unpasteurised milk, cheese, and butter, as shown in Figure 1. The sampling area involved different provinces of the Lombardy region (Figure 2); the sampling size and locations are shown in Figure 3.

Passive sampling involved raw milk (primarily from goats), butter, and unpasteurised milk cheese (goats and cattle) mainly during the period of activity of the alpine pastures (May–September); it combined the Alpine Pasture Plan already established by the Local Official Health Authority and the voluntary self-check plan for Hazard Analysis Critical Control Point (HACCP) by Food Business Operators (FBOs). Raw goat milk samples were also delivered by the National Reference Centre of Milk Quality (IZSLER, Brescia, Italy). Sheep products were not collected because of the poor presence of sheep herds.

### 2.2 Sample preparation

The preparation of specimens varied according to the sample to be analysed and followed an internal procedure that was specifically developed for each matrix.

#### 2.2.1 Cheese

The samples were prepared by mixing 10 g with 30 mL Tris-glycine 1% beef extract (TGBE—composed of Tris base, glycine, beef extract powder, and molecular biology grade water; pH 9.5) and adding 10 μL of process control Mengovirus (recombinant mengovirus-vMC0 strain, ATCC VR-1597™; 10^4^ viral particles/μl). After homogenising with a stomacher for 2 min, a solution of 0.15 mg/mL of proteinase K was added. The samples were then incubated at 37°C for 15 min, followed by 4°C for 15 min. After centrifugation at 8500× *g* for 15 min, the eluate was filtered, and 1 mL was recovered for viral RNA extraction.

#### 2.2.2 Butter

A total of 10 g of sample were spiked with 10 μL of process control Mengovirus (recombinant mengovirus-vMC0 strain, ATCC VR-1597™; 10^4^ viral particles/μl) and incubated at 50°C for 10 min in a water bath to separate the fatty phase. The aqueous phase was recovered and treated with 4:1 (v/v) TGBE buffer (pH 9.5) and 0.15 mg/mL of proteinase K. The mixed sample was incubated at 37°C for 15 min, followed by 4°C for 15 min. After centrifugation at 8500× *g* for 15 min, the eluate was filtered, and 1 mL was collected for viral RNA extraction.

#### 2.2.3 Raw milk

Samples of raw milk were collected in containers containing sodium azide. The preliminary tests demonstrated that the presence of sodium azide in the raw milk tubes did not impact the results of the real-time RT-PCR, as evidenced by the absence of any inhibitory effects (data not shown). This finding was further corroborated by the detection of Mengovirus (recombinant mengovirus-vMC0 strain, ATCC VR-1597™; 10^4^ viral particles/μl), which served as a process control at the final stage of the procedure.

In total, 10 mL of sample was spiked with 10 μL of process control Mengovirus (recombinant mengovirus-vMC0 strain, ATCC VR-1597™; 10^4^ viral particles/μl), and treated with 30 mL TGBE buffer (pH 9.5) and 0.15 mg/mL of proteinase K. The sample was briefly mixed and incubated at 37°C for 15 min and then at 4°C for 15 min. After centrifugation at 8500× *g* for 15 min, the eluate was filtered, and 1 mL was collected for viral RNA extraction.

### 2.3 RNA purification and extraction

Viral RNA was extracted and purified using the NucliSENS^®^ eGENE-UP^®^ system (bioMérieux SA, Marcy-l’Etoile, France) following the manufacturer’s instructions. Viral particles were recovered, concentrated from the matrix, and lysed in an aqueous phase using specific buffers and enzymes, alternating with centrifugation steps.

A positive extraction control Mengovirus (10 μL of recombinant mengovirus-vMC0 strain, ATCC VR-1597™; 10^4^ viral particles/μl) and a negative extraction control were included in each analysis. Following the extraction process, the samples were subjected to a real-time RT-PCR analysis. Only after this stage, extracted RNA was stored at −80°C.

### 2.4 One-step real-time RT-PCR

#### 2.4.1 Method for the detection of TBEV using one-step real-time RT-PCR

RNA was amplified using one-step real-time RT-PCR with specific primers and probes designed for the determination of TBEV in milk and dairy products (Schwaiger and Cassinotti, 2003; Balogh et al., 2012) (Table 1).

**Table 1:** Primers and probes used for the TaqMan^™^ TBEV real-time RT-PCR.

Sequence (5′ ➔ 3′)	Genome positions
Primer F-TBE1: GGG CGG TTC TTG TTC TCC	(11,054 /11071)^a^
Primer R-TBE1: ACA CAT CAC CTC CTT GTC AGA CT	(11,099/11121)^a^
TBE-Probe-WT: TGA GCC ACC ATC ACC CAG ACA CA	(11,073/11095)^a^

a Sequence of TBEV strain neudoerfl (accession number: U27495) (Schwaiger and Cassinotti, 2003).

The reaction was conduted using the RNA UltraSense™ One-Step Quantitative RT-PCR System (Invitrogen, Carlsbad, CA, United States) in a total volume of 20 μL containing 5 μL of UltraSense reaction mix (5×), 1 μL of each primer (12.5 μM and 22.5 μM, Forward and Reverse, respectively), 1 μL of probe (6.25 μM), 0.5 μL of Rox reference dye (50×), 1.25 μL of RNA Ultrasense enzyme mix, and 10.25 μL of DNAse-RNase-free water (Sigma–Aldrich, St. Louis, MO, USA). A total of 5 μL of RNA was added to the reaction mix.

Reverse transcription was performed for 60 min at 55°C; the samples were then incubated at 95°C for 5 min and amplified for 45 cycles of 15 s at 95°C, 1 min at 60°C, and 1 min at 65°C (Schwaiger and Cassinotti, 2003).

The positive amplification control was derived from a positive field sample isolated from a dog exhibiting neurological symptoms. The viral strain belonged to the TBEV-Eu subtype. Negative (DNase-RNase-free water) and positive amplification controls were included in each run.

#### 2.4.2 Method for the detection of Mengovirus using one-step real-time RT-PCR

The primers, probe, reaction conditions, and thermal profile for the detection of Mengovirus are described in ISO/TS 15216–2:2019. RNA extraction efficiencies were calculated according to ISO/TS 15216–2:2019 (ISO, 2019). The required acceptability limit for process control virus recovery was 1%.

## 3 Results

A total of 248 samples from the Lombardy region, namely, 137 cheese, 62 butter, and 49 raw milk, were analysed.

The specific real-time RT-PCR showed that none of the samples tested positive for the presence of the viral RNA.

The TBEV prevalence was 0% in all the sampling provinces; the confidence intervals (95% CIs) calculated using the binomial method based on the beta distribution were as follows: Brescia, 95% CI: 0.00–2.86; Bergamo, 95% CI: 0.00–6.49; Lecco, 95% CI: 0.00–18,53; Sondrio, 95% CI: 0.00–16.84; Como, 95% CI: 0.00–21.86; Varese, 95% CI: 0.00–28.49; and Monza-Brianza, 95% CI: 0.00–84.19.

## 4 Discussion

Alimentary TBEV infections are recognised as a relevant factor contributing to the overall increase in TBE incidence in Europe (Ličková et al., 2021). Foodborne TBE outbreaks have been reported in several endemic countries in Central Europe at a regular frequency, particularly in countries where the consumption of traditional raw milk products is popular (Gonzalez et al., 2022).

When an animal is bitten by an infected tick, the virus can be secreted in low concentrations in milk; nevertheless, products made from unpasteurised milk can be infectious for human consumers (Balogh et al., 2010).

This study was carried out to investigate the presence of TBEV in raw foodstuffs produced by ruminants bred on the Lombard Alps, in areas bordering the endemic Triveneto region.

TBEV may be transmitted from goats via milk where the virus can be isolated for 3–25 days after infection. In cattle, the virus can also be isolated from milk samples, with a viral load detected lower than that observed in sheep and goats (Ličková et al., 2021; Gonzalez et al., 2022; Martello et al., 2022). However, information on infection dynamics in ruminants is relatively scarce, particularly in naturally infected animals.

Foodborne transmission of TBEV occurs when non-pasteurised milk and cheese from viraemic animals (e.g., cows, goats, and sheep), mainly in endemic areas, are consumed.

TBEV is infectious in various dairy products, including yoghurt, cheese, and butter. Indeed, outbreaks have been observed in farming families who consumed unpasteurised dairy products from their own farms (Holzmann et al., 2009; Brockmann et al., 2018; Ličková et al., 2021; Elbaz et al., 2022).

Unlike milk-borne TBE epidemics, there have been no reports of large-scale cheese-borne TBEV infections in humans, except for sporadic cases. This could be attributed to the common practice among pasture producers to manually salt the surface of cheese or place it in brine, which may explain why all cheese products analysed in the present study tested negative for TBEV by real-time RT-PCR.

In fact, TBEV can be inactivated in raw goat cheese through salt treatment (18–25 wt% NaCl) and 72°C pasteurisation (Rónai and Egyed, 2020).

Salting showed an effect in reducing the viral titre together with the pasteurisation of milk but, for the present study, due to the high diversity of dairy products, it would be appropriate to carefully evaluate the process parameters of each product to determine the risk levels for consumers.

In a previous study, the survival of TBEV was observed in heated contaminated milk collected from TBEV experimentally infected goats. The authors observed that the heat treatment at 65°C for 5 min was insufficient for TBEV inactivation, while 3 min at 100°C were the minimal conditions to totally inactivate high viral loads in milk (Balogh et al., 2012).

Other studies evaluated the survival of TBEV in both pasteurised and unpasteurised goat’s milk and cheese that were inoculated with low and high titres of infectious virus. The results showed that both pasteurisation and salt treatment made goat’s milk and cheese safe for consumption (Ličková et al., 2021).

The objective of the study was to gather preliminary data to partially address the current information deficit in the area under investigation. Consequently, passive sampling was conducted in addition to the official Alpine Pasture Plan sampling. Nevertheless, despite the extensive range of milk products subjected to analysis, none tested positive.

It is acknowledged that a limitation of this approach was that it did not guarantee complete uniformity in terms of sampling locations and division into different matrices. However, the advantage was that it expanded the number of samples and matrices analysed, thus providing a preliminary overview of the large area under investigation.

The negative results could have been due to the actual absence of TBEV in collected products. TBEV-free milk, cheese, and butter could have been effectively obtained by healthy animals or with scarce viraemia. A limitation of the study was that no serological investigation was conducted on animals in farms producing the collected samples. Furthermore, the absence of TBEV-positive products may also be attributed to the very low concentration of TBEV in the samples, considering that real-time RT-PCR was previously validated with a limit of detection (LOD) equal to 10^2,8^ TCID_50_/10 g for cheese, 10^2,8^ TCID_50_/10 mL for milk and 10^1,8^ TCID_50_/10 g for butter, respectively (TCID_50_: 50% tissue culture infectious dose on artificially infected cells) and with a recovery rate of process control RNA >1%.

However, it should be considered that TBEV is endemic in areas bordering the Lombardy region (Triveneto to the east and Switzerland to the north). The virus has already spread westward, as confirmed by positive ticks collected in these areas (Carpi et al., 2009; Ventura Spagnolo et al., 2018; Alfano et al., 2020; Garcia-Vozmediano et al., 2020).

Moreover, due to the geographical expansion of ixodid ticks, which are competent vectors of TBEV for humans and animals, as a result of climate change, it would be appropriate to consider implementing a programme of continuous surveillance of raw dairy products, farm animals, and wild ruminants, in addition to monitoring the ticks themselves. This approach could, in fact, facilitate an evaluation of the possible silent circulation of the virus, which could also affect areas currently considered non-endemic (such as the Lombardy region).

The recent human cases reported in Lombardy and other Italian regions highlight the necessity of promoting vaccination among affected individuals and high-risk population groups (e.g., hunters, hikers, and farmers) (Gaffuri et al., 2024) and developing strategies to ensure the early detection of foodborne outbreaks and to prevent subsequent clinical cases.

## 5 Conclusion

This is the first monitoring of food at risk of TBEV transmission in a non-endemic region (Lombardy) with evidence of TBEV circulation (Gaffuri et al., 2024).

Our preliminary results suggest that raw milk and raw dairy products do not pose a significant risk of TBEV transmission to humans in the territory of Lombardy. However, due to the continuous westward spread of ticks responsible for transmitting the virus, foodborne TBEV poses a potential growing threat to consumers and should be monitored continuously.

These should be informed about the potential contamination of unpasteurised dairy products, such as milk, cheese, or butter, to raise awareness of the associated risks.

It is recommended that human and animal healthcare systems implement a continuous monitoring programme to assess the presence of the virus in ticks, wild and domestic ruminants, and dairy products at a high risk of contamination.

In order to assess the actual distribution of TBEV in raw dairy products and to improve food safety, it could be particularly useful to develop a food monitoring plan based on screening results from grazing livestock farms. Furthermore, it could be evaluated to extend the sample collection in both endemic and non-endemic areas, thus increasing the epidemiological power of the study and its findings.

In the future, it is necessary to establish the basis for more structured projects involving various stakeholders who can collaborate effectively (One Health Approach). This will help establish the prevalence of TBEV in the territory, in animals, in food products, and in humans.

## Data Availability

The raw data supporting the conclusions of this article will be made available by the authors, without undue reservation.
